# Factors affecting heat-related diseases in outdoor workers exposed to extreme heat

**DOI:** 10.1186/s40557-017-0183-y

**Published:** 2017-06-29

**Authors:** Jungsun Park, Yangho Kim, Inbo Oh

**Affiliations:** 1 Department of Occupational Health, Catholic University of Daegu, Gyeongsan, South Korea; 2 Environmental Health Center, University of Ulsan College of Medicine, Ulsan, South Korea; 3 Department of Occupational and Environmental Medicine, Ulsan University Hospital, University of Ulsan College of Medicine, 877 Bangeojinsunhwando-ro, Dong-gu, Ulsan, 44033 South Korea

**Keywords:** Acclimation, Heat wave, Tropical night, Heat-related disease, Outdoor, Workers

## Abstract

**Background:**

The objectives of the present study are to: (*i)* evaluate the effect of environmental and metabolic heat on heat-related illnesses in outdoor workers; and *(ii)* evaluate the effect of personal factors, including heat acclimation, on the risk of heat-related illnesses in outdoor workers.

**Methods:**

We identified 47 cases of illnesses from exposure to environmental heat in outdoor workers in Korea from 2010 to 2014, based on review of workers’ compensation data. We also obtained the information on location, time, and work environment of each heat-related illness.

**Results:**

Our major results are that 29 cases (61.7%) occurred during a heat wave. Forty five cases (95.7%) occurred when the maximum estimated WBGT (WBGTmax) was equal to or greater than the case specific threshold value which was determined by acclimatization and metabolic rate. Twenty two cases (46.8%) were not acclimated to the heat. Thirty-seven cases (78.7%) occurred after tropical night (temperature above 25 °C), during which many people may find it hard to sleep.

**Conclusion:**

Personal risk factors such as heat acclimation as well as environmental factors and high metabolic rate during work are the major determinants of heat-related illnesses.

## Background

Workers who are exposed to extreme heat while engaged in strenuous physical activities outdoors have an increased risk for heat stress [1–3]. Exposure to extreme heat can result in several occupational illnesses, including heat stroke, heat exhaustion, heat syncope, heat cramps, heat rashes, and even death [4–10]. Heat can also increase the risk of injuries, because it may lead to sweaty palms, fogged-up safety glasses, and dizziness, and may also reduce brain function, leading to impaired reasoning ability, and thereby create additional hazards [11, 12].

Heat stress will become an increasing problem for outdoor workers, because global temperatures are expected to rise due to the global climate change [2]. It seem likely that global climate change will increase known heat-exposure hazards for workers, especially their severity, prevalence, and distribution, although this has not yet been definitively established [13, 14]. During sudden or prolonged heat waves in urban areas, there are sudden increases in mortality, especially among older individuals who apparently have reduced physiologic reserves [5, 15, 16]. In prolonged heat waves, the mortality rate is higher in the early phase than in the later phase [15, 16]. Previous studies on heat wave in general population have been focused on meteorological factors such as air temperature, humidity, wind speed and radiation. Recent papers reported that personal factors such as heat acclimation [17–19], being older than 60 [20–22], experience of a previous heat-related illness [23, 24], use of certain medications [25, 26], presence of certain concurrent diseases [27, 28], severe obesity [15, 20], and dehydration [29, 30] also increase the risk of heat-related illnesses [18]. However, outdoor workers are exposed to heat stress from the meteorological environment, from exertion, and due to the insulation of clothing during work. These workers may also have additional personal risk factors that increase the risk of heat-related illnesses. However, meteorological, metabolic, clothing, and personal factors affecting heat-related diseases in outdoor workers were scarcely studied [31] although heat strokes were reported in soldiers [32–34], indoor workers [35], and general population [36–40] in Korea. In particular, the personal factors were not studied in outdoor workers in Korea.

The objectives of the present study are to: *(i)* evaluate the effect of environmental and metabolic heat on heat-related illnesses; and *(ii)* evaluate the effect of personal susceptible factors including heat acclimation on heat-related illnesses in Korean outdoor workers, to prevent heat-related illnesses.

## Methods

This is a descriptive study on outdoor workers with heat-related illness. We obtained workers’ compensation data for all of Korean workers who were compensated with heat-related illness including heat stroke, heat exhaustion, heat syncope and heat cramps in outdoor workers such as construction workers, or waste disposal workers during previous 5 years prior to the present study (from 2010 to 2014) from Korea Occupational Safety and Health Agency (KOSHA) [41]. We identified 47 cases of illnesses from exposure to environmental heat in outdoor workers. Obtained data include information on age, sex, diagnosis, occupation classified by major, sub-major, and minor categories, and time of employment of workers, the location and time of these events, and the type of work being performed at the time of events. We classified outdoor workers into workers with or without heavy physical work, which is defined by ISO [19], for example, intense arm and trunk work, carrying heavy material, and pushing or pulling heavily loaded carts.

For identification of meteorological factors associated with heat-related diseases, the hourly dry air temperature and relative humidity of the 3 days prior to each heat-related illness were obtained from the closest meteorological sites (Automatic Weather System (AWS) or Automated Synoptic Observing System (ASOS), operated by the Korea Meteorological Administration). A ‘heat wave’ was defined as a period of 2 or more consecutive days with temperatures of 33 °C or higher [42]. A ‘tropical night’ was defined one in which the lowest nighttime temperature was above 25 °C [42]. Wet-bulb globe temperature (WBGT) was estimated using the meteorological data from AWS and ASOS with Park’s formula [43].

$$ \begin{array}{l}\begin{array}{l}{\mathrm{WBGT}}_{\mathrm{estimated}}\left({}^{{}^{\circ}}\mathrm{C}\right)=\hbox{-} 0.24418+{0.553991}^{\ast}\mathrm{Tw}+{0.455346}^{\ast}\mathrm{Ta}\hbox{-} 0.00217\ast {\mathrm{T}\mathrm{w}}^2+{0.002782}^{\ast }{\mathrm{T}\mathrm{w}}^{\ast}\mathrm{Ta}\hfill \\ {}\begin{array}{l}\mathrm{Tw}={\mathrm{T}\mathrm{a}}^{\ast}\mathrm{a} \tan \left[{0.151977}^{\ast }{\left(\mathrm{RH}+8.313659\right)}^{1/2}\right]+\mathrm{atan}\left(\mathrm{Ta}+\mathrm{RH}\right)\kern0.5em \hbox{--} \kern0.5em \mathrm{a} \tan \left(\mathrm{RH}\hbox{-} 1.676331\right)+{0.00391838}^{\ast }{\left(\mathrm{RH}\right)}^{3/2}\\ {}{}^{\ast}\mathrm{a} \tan \left({0.023101}^{\ast}\mathrm{RH}\right)\hbox{--} 4.686035\end{array}\hfill \end{array}\\ {}\mathrm{Ta};\mathrm{air}\ \mathrm{temperature}\ \left({}^{{}^{\circ}}\mathrm{C}\right),{\mathrm{T}}_{\mathrm{w}};\mathrm{wet}\hbox{-} \mathrm{bulb}\ \mathrm{temperature}\left({}^{{}^{\circ}}\mathrm{C}\right),\mathrm{RH};\mathrm{relative}\ \mathrm{humidity}\ \left(\%\right)\end{array} $$

Personal factors associated with heat-related diseases identifiable from workers’ compensation data were limited to age and acclimatization. Unacclimatized workers were defined as workers with less than a week of period of time from the first outdoor placement of worker to the time of the onset of heat-related disease [18]. Lack of sleep was presumed when tropical night occurred just 1 day prior to the occurrence of heat-related disease in outdoor workers [44–46].

We evaluated 47 workers individually in terms of their exposure to meteorological factors, such as heat wave and tropical night, and personal factor such as acclimatization.

The present study utilized workers’ compensation data, which were formally obtained from KOSHA, and did not include identifiable personal information. This study was exempted from Institutional Review Board review (UUH 2017–04-010).

## Results

We identified 47 outdoor workers (43 males and 4 females) who had illnesses following exposure to environmental heat from 2010 to 2014, based on a review of workers’ compensation data (Table 1). Twenty-six workers (55.3%) suffered from heat-related illnesses in 2013, the worst year. Three of the 47 workers were in their 20’s (6.4%), 4 in their 30’s (8.5%), 13 in their 40’s (27.7%), 15 in their 50’s (31.9%), and 10 in their 60’s (21.3%), and 2 in their 70’s (4.2%). Twenty-three individuals (48.9%) were construction workers, 6 were cleaning and janitorial workers, 4 were agroforestry workers, and the others worked in various different fields. The heat-related illnesses were heat stroke (39 cases), heat cramp (1 case), heat exhaustion (5 cases), and heat syncope (2 cases). There were 11 fatalities.

**Table 1:** Forty-seven compensated cases of occupational heat-related illnesses in outdoor workers in Korea from 2010 to 2014

No.	Heavy exertion	Unaccli-matized	Age	Tropical night	Heat wave	WBGT
1	o	x	41	x	o	31.75
2	o	x	55	x	o	32.59
3	o	x	66	x	o	30.24
4	o	x	29	o	o	30.62
5	o	x	54	o	o	29.9
6	o	x	44	o	o	31.76
7	o	x	49	o	o	30.98
8	o	x	63	o	o	31.29
9	o	x	50	o	o	30.76
10	o	x	29	o	o	32.29
11	o	x	53	o	o	29.0
12	o	x	44	o	o	29.97
13	o	x	46	o	o	29.14
14	o	x	62	o	o	31.89
15	o	o	45	o	o	25.71
16	o	o	35	o	o	30.37
17	o	o	54	o	o	30.28
18	o	o	44	o	o	31.27
19	o	o	56	o	o	29.88
20	o	o	44	o	o	30.77
21	o	o	56	o	o	29.03
22	o	o	52	o	o	30.48
23	o	o	45	o	o	29.31
24	o	o	39	o	o	30.78
25	o	o	39	o	x	25.98
26	o	o	42	o	x	29.29
27	o	o	54	o	x	31.66
28	o	o	51	o	x	30.09
29	o	o	49	o	x	30.76
30	o	o	47	o	x	28.85
31	o	o	64	o	x	29.09
32	o	o	62	o	x	27.46
33	o	o	51	o	x	32.72
34	o	o	66	o	x	29.57
35	o	x	47	o	x	30.59
36	o	x	50	x	x	29.73
37	o	x	60	x	x	30.11
38	o	x	54	x	x	30.39
39	o	x	49	x	x	24.37
40	o	x	60	x	x	23.43
41	o	x	59	x	x	25.87
42	x	x	67	o	o	32.59
43	x	x	56	o	o	30.67
44	x	x	77	o	o	30.53
45	x	o	37	o	o	29.29
46	x	o	51	x	x	30.72
47	x	x	71	o	o	32.45

Our evaluation of the 47 compensated cases of occupational heat-related illness in outdoor workers during 2010 ~ 2014 shows that 29 cases (61.7%) occurred during a heat wave. Forty five cases (95.7%) occurred when the maximum estimated WBGT (WBGTmax) was equal to or greater than the case specific threshold value which was determined by acclimatization and metabolic rate [19]. Twenty two cases (46.8%) occurred in workers who were not acclimatized workers, and 37 cases (78.7%) occurred after a tropical night.

## Discussion

Heat stress is the net heat load to which a worker is exposed from the combined contributions of environmental factors, metabolic heat, and clothing, all of which increase heat storage in the body [17, 18]. The heat load experienced by a worker provokes a physiological response (heat strain), that increases heat loss from the body in an effort to maintain a stable body temperature. This physiological response is not always successful and, when unsuccessful, may result in heat injury and death [17, 18].

The specific environmental factors causing heat stress are high air temperature, minimal movement of air, high humidity, and radiant heat. Physical work contributes to the total heat stress of a job, because metabolic heat increases in proportion to work intensity [19]. The amount, thermal characteristics, and type of clothing worn are also important, because they alter the rate of heat exchange between the skin and the air [47, 48].

In most situations environmental heat exposure can be assessed by WBGT [3, 49]. The WBGT also considers metabolic heat and insulation from clothing [17, 19]. The metabolic contribution to the heat load of the worker can be estimated based on the extent of physical exertion (light, moderate, or heavy) [19]. Lower WBGT thresholds should be applied to workers when they perform heavy work than light work. WBGT limits assume the person is wearing a conventional one-layer work clothing ensemble, consisting of not more than a long-sleeved work shirt and trousers (or the equivalent) [50]. Thus, a lower WBGT limit should be applied to workers who wear clothes with lower air and vapor permeability or that provide greater insulation than conventional clothing [19, 48, 51–53].

In addition to environmental heat, metabolic heat, and clothing insulation, additional personal factors affect the risk of heat-related illness. These include heat acclimation [17–19], dehydration [29, 30], unique medical characteristics [23, 24, 26], and overall health status at the time of exposure to heat stress [28, 29].

The present study had several important findings. First, 61.7% of heat-related events occurred during periods when there was a high dry bulb air temperature (heat wave), and 95.7% occurred when the temperature exceeded the WBGT limit. These findings suggest that the WBGT limits can predict heat-related illness better than air temperature [54]. The WBGT-based limit (but not the dry bulb temperature) is based on other environmental factors, such as water vapor pressure, movement of air, and radiant heat, and this limit can be adjusted according to metabolic heat and the type of clothing [49, 55].

Second, 46.8% of the heat-stressed workers in our study were not acclimatized to a high temperature. This finding is compatible with the results of Arbury et al. [56]. They presented 22 cases of heat-related illness or death in a population of workers. In most cases, the employers had no program to prevent heat illness, or the program was deficient. Acclimatization was the most commonly missing program element, and the element most clearly associated with worker death. Appropriate repeated exposure to elevated heat stress causes a series of physiological adaptations -- acclimatization -- in most people, in which the body becomes better able to cope with heat stress. An acclimatized worker can tolerate greater heat stress [25, 57–59]. Different exposure limits to heat are applied, depending on the acclimation of each individual. The current National Institute of Occupational Safety and Health (NIOSH) Recommended Alert Limits (RALs) are for unacclimatized workers and the Recommended Exposure Limits (RELs) for acclimatized workers [60, 61]. Occupational Safety and Health Agency (OSHA) defines the WBGT–based limit differently for acclimatized and unacclimatized workers [47], as does the American Conference of Governmental Industrial Hygienists (ACGIH) threshold limit value (TLV) [17], the American Industrial Hygiene Association [62], and the international standard organization [19]. Acclimatization to work in hot, humid environments provides adaptive benefits [25], so an acclimatization plan should be implemented at all workplaces where workers are exposed to heat.

Third, a tropical night on the previous day was associated with 78.7% of heat-related illnesses, but high daytime temperature (heat wave) was associated with 61.7% of heat-related illnesses. The most common consequence of the warm nights on health is their impact on sleep of workers. Heat can cause disturbances and sleep deprivation by thermoregulation processes [44]. Higher than the comfort temperature can influence sleep loss and the reduction of rapid eye movement (REM) and slow-wave sleep (SWS) phases [45, 46]. Sleep disorders occur more frequently, as the risk of death during heat waves, in people with advanced age [44, 63]. During tropical nights, many people may not sleep well, and this lack of sleep may increase the risk for heat-related illness.

Finally, 87.2% of 47 outdoor workers did heavy physical work. This finding showed that metabolic heat is an important factor to increase the risk of heat-related illness in addition to environmental heat. Older outdoor workers were more likely to increase the risk for heat-related illness although they did not perform heavy physical work.

Taken together, personal factors, as well as environmental factors and metabolic heat, are the major determinants of heat-related illnesses. Moreover, many factors other than heat acclimation or lack of sleep may make individuals more susceptible to heat-related illnesses. Recent papers reported that being older than 60 [20–22], experience of a previous heat-related illness [23, 24], use of certain medications [25, 26], presence of certain concurrent diseases [27, 28], severe obesity [15, 20], and dehydration [29, 30] also increase the risk of heat-related illnesses [18].

**Fig. 1:**
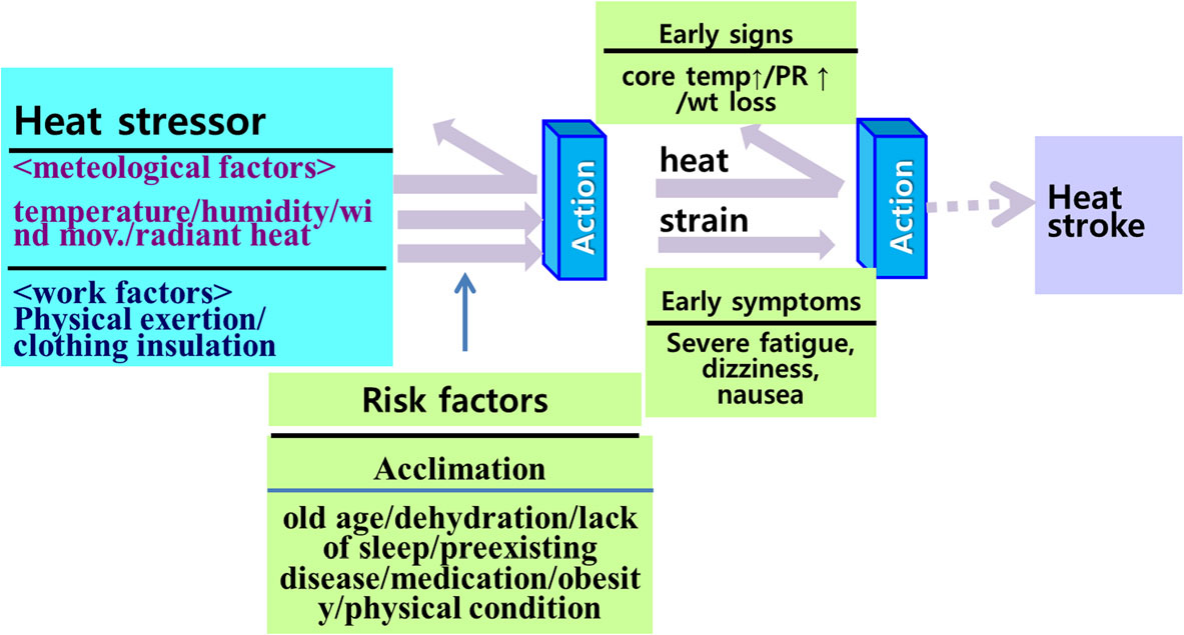
Proposed mechanism of heat-related illnesses

Our research led to a model for the development of heat-related illnesses (Fig. 1). When excessive environmental and/or metabolic heat stress are combined with personal risk factors, physiological responses (heat strain) that promote transfer of heat back into the environment to maintain core body temperature cannot adequately maintain body temperature [64]. This leads to increased core temperature and pulse rate, and decreased body weight due to dehydration [17]. Without early interventions in an individual with early signs of heat-related illness, the heat strain can become harmful and the worker may become a heat casualty [18].

A limitation of the present study is that we only considered two personal risk factors: heat acclimation and lack of sleep. We were not able to obtain information on additional personal factors that affect the risk of heat stress such as dehydration, experience of a previous heat-related illness, use of certain medications, presence of certain concurrent diseases, and overall health status at the time of exposure to heat stress from the data base.

## Conclusion

Personal risk factors, environmental factors, and metabolic heat are the major determinants of heat-related illnesses. All workplaces should implement acclimatization plans for workers who are exposed to heat. Managers should also check the status of workers, such as lack of sleep, dehydration, and consumption of alcohol before work.

## Abbreviations

ACGIH: American Conference of Governmental Industrial Hygienists
ASOS: Automated Synoptic Observing System
AWS: Automatic Weather System,
NIOSH: National Institute of Occupational Safety and Health
OSHA: Occupational Safety and Health Agency
RAL: Recommended Alert Limit
REL: Recommended Exposure Limit
REM: Rapid eye movement
RH: Relative humidity
SWS: Slow-wave sleep
T_a_: Air temperature
TLV: Threshold limit value
T_w_: Wet-bulb temperature
WBGT: Wet-bulb globe temperature
